# Editorial: Exploring clinical outcomes and treatment predictors in septic arthritis

**DOI:** 10.3389/fmed.2026.1855262

**Published:** 2026-05-19

**Authors:** Serban Dragosloveanu, Oana Săndulescu, Eric L. Smith, Javad Parvizi

**Affiliations:** 1 Faculty of Medicine, The “Carol Davila” University of Medicine and Pharmacy, Bucharest, Romania; 2 Department of Orthopaedics, “Foisor” Clinical Hospital of Orthopaedics, Traumatology and Osteoarticular TB, Bucharest, Romania; 3 National Institute for Infectious Diseases “Prof. Dr. Matei Balş”, Bucharest, Romania; 4 Society for Research and Innovation in Infectious Diseases, Bucharest, Romania; 5 Infection Science Forum, Bucharest, Romania; 6 Department of Orthopaedic Surgery, New England Baptist Hospital, Boston, MA, United States; 7 International Joint Center, University of Acibadem, Istanbul, Türkiye

**Keywords:** antimicrobial resistance, biomarkers, comorbidities, periprosthetic joint infection, prognosis, risk factors, septic arthritis, treatment outcomes

Septic arthritis (SA) and periprosthetic joint infection (PJI) are challenging complications characterized by substantial morbidity, risk of joint degradation, and significant mortality. While surgical techniques, antimicrobial therapy, and diagnostic tools have known major progress in the last decade, numerous hurdles remain related to accurate diagnosis and optimal management, especially in regard to comorbidities, evolving pathogen profiles, and heterogeneous clinical presentations (1, 2). Recent guidelines and consensus definitions have brought significant improvements to the diagnosis structure by promoting combined methods over single-test approaches but a real gold standard is yet to be defined (3). The present Research Topic brings together a collection of studies that address key aspects of diagnosis, treatment optimization, and prognostic stratification in septic arthritis and related joint infections.

A main aspect becoming evident from this Research Topic is the ongoing challenge represented by accurate and prompt diagnosis and the scientific interest in standardizing diagnostic pathways. Traditional microbiological methods are essential for etiological diagnosis, but their limitations are increasingly recognized. Wu et al. investigated whether extending culture duration improves diagnostic yield in PJI and found that prolonged incubation did not significantly increase culture positivity or improve clinical outcomes. These findings question the clinical utility of extended culture protocols and point out the need for more efficient diagnostic strategies. However, while extended incubation may not be helpful for acute or typical infections, it may remain a valid strategy for slower-growing organisms and bacteria adapted to biofilm in chronic infections or cases previously exposed to antibiotics (4, 5).

At the same time, efforts to improve diagnostic precision through biomarker development are demonstrated by Cao et al., who examined ratio-based indicators derived from prealbumin. Their study showed that markers such as CRP/prealbumin (CPR) and combined indices have good diagnostic performance, particularly in some patient subgroups such as those living with diabetes. These results align with the emerging concepts of integrating nutritional and inflammatory parameters into diagnostic algorithms in order to obtain higher sensitivity and specificity in differentiating infection from aseptic conditions. Several recent studies also supported the potential predictive role of hypoalbuminemia and poor nutritional status for adverse outcomes in PJI, but underline that these instruments should be used as risk predictors rather than diagnostic elements (6, 7).

In addition to improving diagnostic accuracy, these biomarker-based approaches extend into the prognostic domain. Another key dimension explored in this Research Topic is the identification of prognostic factors and predictors of treatment outcomes. Huang et al. investigated the role of inflammatory and nutritional markers in predicting failure of prosthesis removal and antibiotic spacer implantation. Their findings indicate that increases in markers such as CRP and the CRP/albumin ratio above certain thresholds are strongly associated with treatment failure, suggesting their potential application in preoperative risk stratification. These results also support the concept that systemic inflammation and nutritional status significantly impact the host response and recovery in infectious conditions (8).

While biomarker-based approaches contribute to the diagnosis and prognostic stratification approach, a complete understanding of SA also requires consideration of microbiological and host-related determinants. In this context, Straub et al. explore pathogen distribution and antimicrobial resistance trends in native joint septic arthritis. The authors confirm the predominance of *Staphylococcus aureus*, while also demonstrating relatively low rates of methicillin-resistant strains and stable proportions of gram-negative organisms. Moreover, the study reveals evolving resistance patterns with significant variability in clindamycin susceptibility. This is a very relevant result in supporting empirical therapeutic decisions, but it should not be generalized, as local surveillance of antibiotic resistance is indispensable (9). Also, the treatment of SA and PJI should remain anchored in local microbiology patterns and not assumptions based on older or foreign patient cohorts (10).

At the same time, the importance of host-related factors is further demonstrated in the papers of Khudair et al. and Akhtar et al., both of which reveal the significant impact of comorbidities on the prognosis of septic arthritis. Conditions such as diabetes mellitus, rheumatoid arthritis, and chronic kidney disease are consistently associated with increased susceptibility to infection, delayed diagnosis, and poorer clinical outcomes. These comorbidities not only impair immune function but also complicate surgical decision-making and antimicrobial management, which leads to higher rates of treatment failure and mortality (9).

Ultimately, translating these insights into effective clinical management requires careful optimization of antimicrobial therapy. Zhang et al. evaluated the role of adjunctive rifampin in the treatment of staphylococcal PJI and reported no significant improvement in treatment success rates, while noting a higher incidence of adverse drug events. This finding challenges the widespread assumption of rifampin's universal benefit in biofilm-associated infections and draws attention to the importance of individualized antimicrobial strategies based on patient characteristics and risk profiles (11).

Taken together, the studies in this Research Topic demonstrate the multifactorial nature of SA and PJI and enforce the concept that diagnosis is evolving from using conventional culture techniques toward more complex approaches that incorporate biomarker profiles and host factors. However, therapeutic strategies must also take efficacy and safety into consideration, and clinical outcomes can be further improved through the identification of reliable prognostic indicators, which provide the opportunity to personalize treatment pathways. This collection of papers suggests several directions for future research. First, there is a need to standardize diagnostic flows while integrating microbiological, biochemical, and clinical parameters. Second, further investigation into novel biomarkers and their potential validation in different patient populations is essential for enhancing diagnostic precision. Third, antimicrobial management must remain a priority, with ongoing surveillance of resistance patterns and careful assessment of adjunctive therapies. Finally, the role of comorbidities and patient-specific factors should be further addressed, supporting the development of individualized management strategies.

In conclusion, this Research Topic provides a detailed overview of current challenges and emerging solutions in the management of SA and PJI. These contributions advance our understanding of diagnostic innovation, therapeutic evaluation, and prognostic assessment with clear perspectives that continued interdisciplinary research and clinical collaboration will be crucial to translating these insights into improved patient care and outcomes.
